# eHealthism in inclusion health: a qualitative longitudinal study with women experiencing homelessness

**DOI:** 10.1186/s12939-026-03061-0

**Published:** 2026-10-10

**Authors:** Sophie Nadia Gaber, Elisabet Mattsson, Anna Klarare, Jenny Eriksson Lundström, Penny Rapaport, in collaboration with the Women’s Advisory Board for Inclusion Health

**Affiliations:** 1 Department of Women’s and Children’s Health, CIRCLE – Complex Intervention Research in Health and Care, Uppsala University, Dag Hammarskjölds väg 14B, Uppsala, 751 05 Sweden; 2 Faculty of Brain Sciences, Division of Psychiatry, University College London, London, England; 3 Department of Healthcare Sciences, Marie Cederschiöld University College, Stockholm, Sweden; 4 Connell School of Nursing, Boston College, Boston, USA; 5 Department of Informatics and Media, Uppsala University, Uppsala, Sweden; 6 Ersta Möjlighet, Stockholm, Sweden

**Keywords:** Women’s health, Digital health, eHealth, Healthism, Homelessness, Inclusion health, Narrative

## Abstract

**Background:**

While inclusion health approaches are increasingly recognised in research, policy, and practice as essential to addressing health inequities and social exclusion, there is limited understanding of how women experiencing homelessness engage with digital health. This represents a salient knowledge gap, given the growing interest in digital health to improve access, flexibility, and patient empowerment for marginalised populations. This study aimed to explore: (1) how women experiencing homelessness access and use digital technologies to support their health and wellbeing in everyday life; and (2) how their engagement with digital health is shaped by, and shapes, their interactions with health and social care systems, as well as with third-sector organisations.

**Methods:**

This qualitative longitudinal study gathered data through narrative interviews and participant observations with nine women experiencing homelessness in London, England. Data were analysed using a three-phase phenomenological-hermeneutic approach inspired by Ricoeur’s narrative and interpretation theory. Women with lived experience of homelessness contributed to the study design and recruitment processes, co-produced study materials, supported the analysis and interpretation of findings, and co-produced recommendations.

**Results:**

Using eHealthism as an interpretive lens, our analysis identified three themes: (1) Taking care of my health on my own terms – with or without digital tools; (2) When digital tools are not enough – I still need support; and (3) Digital health can work – but it’s not the same as seeing someone face-to-face.

**Conclusions:**

Based on the findings, we propose a three-layer conceptual framework of exclusion mechanisms in eHealthism, along with corresponding recommendations for inclusion health. Together the individual, relational, and contextual layers of exclusion mechanisms challenge assumptions of individual responsibility and universal digital empowerment, highlighting the need for meaningful choice in hybrid care and for ongoing, trauma‑informed relational support. Addressing these complexities is essential for developing inclusive digital health strategies that advance health equity for women experiencing homelessness and other groups at risk of social exclusion.

**Supplementary Information:**

The online version contains supplementary material available at https://doi.org/10.1186/s12939-026-03061-0.

## Background

Homelessness is increasing in most high-income countries [1], with growing evidence highlighting how gendered vulnerabilities [2, 3] and digital inequities [4, 5] shape the experiences of people experiencing homelessness. Among high-income countries, the United Kingdom (UK) has one of the highest overall rates of homelessness including women experiencing homelessness [1], though women’s experiences are under-reported [2, 6]. In the UK, homelessness is shaped by structural factors such as housing shortages, austerity-driven service constraints, and reliance on a mixed system of statutory (e.g., National Health Service [NHS] and local authorities) and third-sector provision.

*Inclusion health* has emerged as a service, research, and policy agenda aimed at redressing the severe and overlapping health and social inequities encountered by people at risk of social exclusion [2, 7]. This includes people experiencing homelessness, as well as other groups such as those with lived experience of sex work, substance misuse, prison and the criminal justice system, and migration-related vulnerabilities (hereafter collectively referred to as “inclusion health groups”) [8]. While digital health is not inherently an inclusion health approach, it is increasingly discussed as a potential means of supporting more accessible, flexible, and responsive service delivery, particularly when implemented with explicit attention to digital inclusion and equity [9, 10]. However, these benefits are not achieved automatically, and it is important to consider inclusion health from the outset in developing digital health interventions. Digital technologies are becoming increasingly embedded in health and social care. In the UK, this transformation has been accelerated through NHS-led digital strategies and the expansion of remote and online service delivery, particularly following the COVID-19 pandemic [9, 10]. Understanding how inclusion health groups engage with these technologies is therefore imperative [12].

From an inclusion health perspective, digital health is not simply a technological innovation but a potential determinant of equitable access to care. While digital technologies may help overcome barriers to healthcare access, women experiencing homelessness often experience intersecting forms of disadvantage, including poverty, trauma, migration-related vulnerabilities, violence, and digital exclusion [2–5]. Consequently, understanding digital health engagement among women experiencing homelessness requires attention to both digital access and the broader social and structural determinants that shape health equity [7, 12].

Despite the increasing digitalisation of health and social care, accelerated by the COVID-19 pandemic [13], inclusion health groups’ experiences with digital health and its alignment with their complex needs remain under-researched. Existing digital health research has largely focused on homelessness in general [14–16], without gender-disaggregated data, or has been siloed around other sub-groups such as youth [17, 18] or older adults [19, 20]. There has been a growing trend toward co-producing digital health research with people who have lived experience of homelessness [21], including women [22], as experts by experience.

While there is no consensus definition, digital health generally refers to the use of information and communication technologies (ICTs) to support and enhance various aspects of healthcare, from prevention and diagnosis to treatment, monitoring, and management of health [23]. Digital health encompasses a range of related domains, including eHealth (i.e., ICT-based health systems and communication), telehealth (i.e., remote delivery of healthcare), and mHealth (i.e., mobile and wearable health tools and services), as well as emerging technologies such as artificial intelligence. Together, digital health offers increasingly personalised and on-demand care options [16, 24]. Rather than imposing predefined categories (e.g. apps, websites, or telehealth), this study adopts an inductive approach to understanding digital health, recognising that participants often experienced and described digital health resources in fluid and overlapping ways that did not align with discrete technological classifications.

Digital health may help address some of the barriers commonly encountered by inclusion health groups, including difficulties accessing in-person services [11]. These barriers include logistical and service-level challenges, many of which are reflected in UK service provision, including pressures on primary care access, fragmented service pathways, and variability in local authority and third-sector support [25, 26]. They also include individual and structural-level barriers, such as stigma [3, 27, 28], lack of identification [29], no fixed address [15, 29], and difficulties navigating fragmented [27], underfunded and overstretched services [3].

The health and social needs of people experiencing homelessness are complex and often unmet by existing services [3, 26]. Mortality rates among women in inclusion health groups in high-income countries, including those experiencing homelessness, are estimated to be up to twelve times higher than those of women in the general population, and significantly higher than those of men from equivalent groups [7]. Increased mortality risk among people experiencing homelessness is linked to causes that could be avoided with timely and effective access to healthcare [30, 31]. Infectious diseases, cardiovascular and respiratory conditions, and mental health and substance use disorders contribute substantially to excess morbidity among inclusion health groups, while mortality is particularly elevated from injury, poisoning, and other external causes [2, 7]. Studies also indicate high rates of autism [32], Alzheimer’s disease and related dementia (ADRD) [33], and cognitive impairment [34] among people experiencing homelessness, highlighting the importance of considering neurodiversity and accessibility in digital health design and implementation.

Research on digital technologies and inclusion health has focused on access, particularly device access and usage patterns. A systematic review among high-income countries found that between 53% and 100% of people experiencing homelessness owned a mobile phone or smartphone, with similar rates between men and women [15]. Yet, their capacity to use online consultations, health apps, or electronic appointment systems depends not only on internet or device access, but also on digital confidence [21], trust in systems [15, 35], and access to stable, private environments to use digital technologies [24]. Studies indicate that telehealth for mental healthcare is generally perceived positively and considered acceptable for different groups experiencing homelessness [36], including youth [17] and older adults [20]. However, evidence from digital health interventions for healthcare more broadly is mixed. For example, a pilot randomised-controlled trial found mixed results for a telehealth (text messaging) intervention to increase patient engagement in a homelessness clinic [14], suggesting that positive perceptions may not directly translate into sustained use or improved outcomes. Despite efforts to promote digital inclusion [12, 13], many women experiencing homelessness continue to experience restricted, disrupted, or conditional social rights and access to digital technologies [4, 5], limiting their capacity to manage health, seek support, or ensure personal safety [15].

Digital-specific barriers, including limited digital- [14, 16] or digital health literacy [36], inadequate internet infrastructure [15, 16], and inequitable device access [15, 21], risk exacerbating disparities and digital exclusion. These challenges are compounded for women experiencing homelessness by intersecting vulnerabilities [3], including exposure to sexual and domestic violence and abuse, stigma, persistent poverty [2], trauma [37], and competing priorities related to meeting immediate needs for shelter, food, safety, and daily survival [4, 26]. These intersecting vulnerabilities can intensify digital exclusion by limiting access to devices and stable connectivity, undermining digital confidence and trust, and constraining the time, privacy, and stability needed to engage with digital technologies. Thus, digital health may benefit from adopting inclusion health approaches characterised by innovative interventions that “meet clients where they’re at” and recognise the intersecting vulnerabilities and needs of women experiencing homelessness and other inclusion health groups [24]. Recent UK policy initiatives have emphasised the importance of digital inclusion in reducing health inequalities [9, 10]; however, there remains limited evidence on how digital health services meet the needs of women experiencing homelessness and other inclusion health groups.

Third-sector (e.g., voluntary and charitable) organisations play a critical role in bridging service gaps by offering housing, basic support [3], and increasingly, digital resources such as internet-enabled devices as well as ICT training for both staff and service users [35]. While some studies have examined how people use digital technologies to support health information-seeking, service navigation, and social connection [15, 16], further research is needed to explore how digital health is embedded in women’s everyday lived experiences, particularly in relation to interactions with health and social care services and third-sector organisations. In summary, despite increasing attention to digital health as a potential strategy to meet the needs of inclusion health groups, there remains a critical gap in understanding how women experiencing homelessness in the UK and similar high-income settings access and use digital technologies to support their health and wellbeing in everyday life. Addressing this gap is essential to inform the design of inclusive, accessible, and contextually appropriate digital health services and policies.

## Objectives

While inclusion health approaches are increasingly recognised across research, policy, and practice as essential for addressing health inequities and social exclusion [2, 7, 8], little is currently known about how women experiencing homelessness engage with digital health. As digital technologies become increasingly embedded within health and social care systems, understanding how inclusion health groups access, navigate, and experience digital health is important to help ensure that digital innovation promotes rather than exacerbates existing health inequities. However, there remains limited understanding about how digital technologies are accessed and used within the context of their everyday lives and intersecting vulnerabilities. This qualitative longitudinal study sought to address this gap by conducting narrative interviews and participant observations with women experiencing homelessness. We aimed to explore: (1) how they access and use digital technologies to support their health and wellbeing in everyday life; and (2) how their engagement with digital health is shaped by, and in turn shapes, their interactions with health and social care systems, as well as with third-sector organisations.

## Theoretical perspective

This study used a phenomenological-hermeneutic approach to elucidate how women experiencing homelessness access and use digital health in their everyday lives. To interpret the participants’ narratives, we have drawn on the concept of *Healthism* [38] and propose a novel extension, *eHealthism*.

Crawford (1980) defined Healthism as “the preoccupation with personal health as a primary – often *the* primary – focus for the definition and achievement of well-being; a goal which is to be attained primarily through the modification of lifestyles, with or without therapeutic help” [38, p. 368]. As such, health becomes not only a personal goal but a responsibility, with expectations for individuals to continually monitor, improve, and optimise their own health and wellbeing through appropriate behaviours, choices, and attitudes [39].

Scholars have critiqued Healthism’s focus on individual responsibility [38] and its neglect of intersecting social and structural determinants of health which disproportionately burden inclusion health groups [39, 40]. For women experiencing homelessness, this highlights tensions between expectations for self-care and the realities of housing instability, exclusion, trauma, and gendered vulnerabilities [2, 37, 41]. The World Health Organization (WHO) has called for greater recognition of human rights, gender equality, and equity in self-care interventions, as well as recognition of how existing practices of self-care are embedded in people’s everyday lives and contexts [42].

As health systems increasingly adopt digital approaches to service delivery [9, 10], individuals are encouraged to access information, communicate with providers, monitor health, and manage care through digital technologies. At the same time, digital health resources such as patient portals, health applications, remote consultations, and self-monitoring technologies are becoming embedded within everyday health practices. These developments raise questions about whether expectations of responsible self-care are increasingly being enacted through digital engagement. While Healthism reflects the moralisation of personal health, eHealthism extends this logic into the digital domain. We define eHealthism as the normative expectation, and increasingly, the social imperative, to engage with digital health as a demonstration of responsible self-care, regardless of a person’s social, economic, political, or technological context and resources.

Within an eHealthist framework, digital health engagement represents self-discipline and virtue [39]. Conversely, non-use or disengagement may be interpreted as personal deficit, irresponsibility, or non-compliance [39]. Such framings obscure material, relational, and contextual barriers to digital health, including disparities in access, literacy, trauma histories, institutional mistrust, and housing precarity [15], and risk further excluding women experiencing homelessness.

Building on earlier literature [39], we propose that eHealthism positions individuals as both consumers and active users of digital health, encouraging investment of time, effort and personal resources in engaging with digital health to optimise health and wellbeing. This emphasis on individual responsibility may exclude those who cannot afford or are unable to navigate digital health [15, 39], such as women experiencing homelessness, thus reinforcing socioeconomic, gender, and digital divides [13]. Moreover, Crawford’s conceptualisation of Healthism highlights the so-called “medicalisation of everyday life” [38], where behaviours and routines (e.g., diet, exercise, socialising, or stress management) are considered to either optimise or hinder health and wellbeing. This is relevant to eHealthism due to the increasing proliferation of digital self-monitoring and self-management into everyday life routines [39].

eHealthism is particularly relevant for examining the experiences of women experiencing homelessness because expectations of digital self-management and personal responsibility may be especially difficult to realise in the context of housing instability, poverty, trauma, digital exclusion, and fragmented access to services. By drawing attention to these tensions, eHealthism provides a lens through which to examine how broader structural constraints shape engagement with digital health.

For the purposes of this study, eHealthism is used as an interpretive lens to explore how digital health is navigated, negotiated, and at times resisted in participants’ everyday lives. This approach builds on a Ricoeur-inspired narrative methodology [43, 44], which informs the interpretation of lived experience throughout the analysis.

## Methods

### Study design

This study is part of a broader cross-national research programme focused on inclusion health with women experiencing homelessness in the UK and Sweden. The present study was conducted in the UK in collaboration with healthcare, public health, and social care services, including NHS services, third-sector organisations (non-profit, non-governmental organisations), academic institutions, and women with lived experience of homelessness as research partners. Table 1 presents an overview of the study timeline and activities. For the present study, we used a qualitative longitudinal design over 16 months (December 2022 - April 2024), employing narrative interviews and participant observations. This approach is well-suited to elucidate how behaviours, experiences, and social norms unfold over time [45], particularly in inclusion health groups such as women experiencing homelessness who may benefit from sustained contact while maintaining confidentiality [41, 46]. We adhered to the Standards for Reporting Qualitative Research (SRQR) (Supplementary file 1) [47].

**Table 1:** Overview of study activities and timeline

Time period	Activity	Participants
Pre-study (2022)	Prioritise focus of grant application, consult on ethics application.	Women’s Advisory Board for Inclusion Health and research team.
	Co-develop participant information about study and recruitment materials.	Women’s Advisory Board for Inclusion Health and research team.
December 2022	Study start-up, contact with recruiting third-sector organisation and 20 service users initially approached to participate in the study based on eligibility criteria.	Third-sector recruiting organisation and research team.
Round 1 data collection (January 2023)	Narrative interviews and participant observations of digital health use during interviews.	Participants (i.e., 8 women experiencing homelessness) and data collector.
	Participant observations of the participants’ interactions with ICT facilitators during weekly ICT classes.	Participants (*n* = 4), ICT facilitators (*n* = 2, staff employed at the recruiting third-sector organisation), and data collector.
	Member-checking discussions.	Participants and data collector. Women’s Advisory Board for Inclusion Health and data collector (in discussions with broader research team).
Round 2 data collection (May 2023)	Narrative interviews and participant observations of digital health use during interviews.	Participants (same as round 1, *n* = 8) and data collector.
	Participant observations of the participants’ interactions with ICT facilitators during weekly ICT classes.	Participants (*n* = 4), ICT facilitators (*n* = 2), and data collector.
	Member-checking discussions.	Participants (*n* = 8) and data collector. Women’s Advisory Board for Inclusion Health and data collector (in discussions with broader research team).
Round 3a data collection (November - December 2023)	Coinciding with the holiday period, recruiting third-sector organisation advised dividing round 3 data collection into two opportunities for participation. 5 participants took part in both round 3a and 3b. Narrative interviews and participant observations of digital health use during interviews.	Participants (4 of the same participants from rounds 1 and 2, with one additional UK-born participant providing a unique perspective to promote information power, *n* = 5), and data collector.
	Participant observations of the participants’ interactions with ICT facilitators during weekly ICT classes.	Participants (*n* = 4), ICT facilitators (*n* = 2), and data collector.
	Member-checking discussions.	Participants (*n* = 5) and data collector. Women’s Advisory Board for Inclusion Health and data collector (in discussions with broader research team).
Round 3b data collection (February-April 2024)	Narrative interviews and participant observations of digital health use during interviews.	Participants (*n* = 9).
	Participant observations of the participants’ interactions with ICT facilitators during weekly ICT classes.	Participants (*n* = 4), ICT facilitators (*n* = 2), and data collector.
	Member-checking discussions, co-construction of recommendations.	Participants (*n* = 9) and data collector. Women’s Advisory Board for Inclusion Health and data collector (in discussions with broader research team)
Post-data collection	Writing up manuscript, option to read manuscript (or excerpts/ oral lay summary by data collector, depending on preferences and capacity).	Participants (*n* = 9) and data collector. Women’s Advisory Board for Inclusion Health and data collector (in discussions with broader research team).

To foster an equity-promoting approach, women with lived experience of homelessness contributed throughout the study, this included both research participants and members of the Women’s Advisory Board for Inclusion Health. The narrative methodology was intentionally selected to foreground participants’ expertise and lived experience through open-ended interviews and observations. Participants shaped the direction of the interviews, determined which aspects of their experiences to discuss, and chose the digital health activities they wished to demonstrate and reflect upon.

Participants were actively involved in the co-construction of their narratives and in identifying salient topics and emerging themes. These interpretations were developed iteratively through discussions between participants and the data collector and were further informed through ongoing collaboration with members of the Women’s Advisory Board for Inclusion Health. The longitudinal design, together with close collaboration with the recruiting third-sector organisation, enabled the data collector to spend time on-site, fostering rapport and trust while creating opportunities for coherence checking and collaborative sense-making (i.e., member checking) between interviews.

The Women’s Advisory Board for Inclusion Health is described in earlier studies [48]. Founded in 2020, it includes women with lived experience of different types of homelessness who attend workshops weekly and are paid per hour based on guidance from the National Institute for Health and Care Research (NIHR) [49]. However, payment guidance was subsequently updated following the completion of data collection. The number of members varies but typically includes approximately eight women. To promote possibilities for sharing power, members prioritised the topic of digital health for the original grant application and contributed to the study design and recruitment processes, co-produced study materials, supported the analysis and interpretation of findings, and co-produced the recommendations. Additionally, their contributions are recognised as co-authors.

### Setting

The study was conducted at a London-based third-sector organisation (registered charity) supporting women experiencing homelessness. This organisation offers hostel accommodation, resettlement support, and at the time of the data collection, a 24-hour, year-round drop-in centre with no referral required. During the data collection period, the organisation supported women aged 18 to 75 years, with over half born outside the UK, including 25% from East Africa. The drop-in centre provided free activities, including weekly ICT, knitting, karaoke, art therapy, and fitness classes. ICT classes were open to all service-users and held twice daily for two hours per session in a classroom with 10 Microsoft PCs and space for personal digital devices.

### Recruitment

Recruitment was guided by the principle of information power [50, 51]. In qualitative research, information power indicates that the more information the sample holds that is relevant for the study, the lower the number of participants needed. Consistent with Malterud et al.’s [50] concept of information power, sample adequacy was assessed in relation to five dimensions: (1) study aim, (2) participant specificity, (3) use of established theory, (4) quality of dialogue, and (5) variation. Information power was strengthened through (1) the study’s focused aim; (2) the high specificity of participants, grounded in their lived experiences of homelessness and digital health resource use; (3) the use of established theory through a phenomenological-hermeneutic approach informed by the concept of Healthism and extended through eHealthism; (4) the quality of dialogue, supported by longitudinal engagement and rapport-building between the researcher and participants; and (5) variation achieved through rich narrative data reflecting diverse intersectional experiences and perspectives.

Staff at the third-sector organisation identified eligible women based on the following inclusion criteria: aged 18 years or older, lived experience of homelessness in England, ability to communicate in English, capacity to give informed consent, and some use of everyday digital technologies (e.g., phone, smartphone, computer). To prioritise the safety and wellbeing of participants, women who appeared to be experiencing severe anxiety, distress, or exhibiting violent behaviours were not invited to take part. This decision was made in consultation with staff to ensure appropriate support and avoid causing further harm.

Recruitment materials were co-produced with staff and an advisory board of women with lived experience of homelessness (the Women’s Advisory Board for Inclusion Health). Staff introduced the study to potential participants, shared verbal and written information, and arranged meetings with the data collector (SNG) to enable them to ask questions and discuss the study. All recruitment and data collection occurred at the third-sector organisation.

### Participants

In total, 20 women were approached about the study based on them matching the eligibility criteria and eight were initially recruited to participate. As the initial eight participants were born outside the UK, an additional participant was recruited later in the study to enhance variation within the participant group and contribute a perspective informed by longstanding familiarity with UK health and social care systems (see Table 1 for details). Participants were purposively sampled to reflect a diversity of experiences based on the European Typology of Homelessness and Housing Exclusion (ETHOS), as shown in Table 2 [52]. Some participants lived temporarily in the third-sector organisation’s accommodation during the study. Based on earlier guidance from the NIHR [49] and discussions with the recruiting third-sector organisation, participants received a £10 grocery voucher after each interview.

**Table 2:** Baseline sociodemographic characteristics of women experiencing homelessness (*n* = 9)

ID	Age	Ethnicity	Country of birth by region	Highest level of education	Employment status	Housing situation	Contact with family and/or relatives	Physical health issues (including comorbidities)	Mental health issues
Evelyn	68	Black other	Latin America and the Caribbean	Primary	Unemployed/ Unable to find work	Hostel	Yes	Allergies, asthma	None reported
Isabel	46	White other	Europe	No formal education	Unemployed/ Unable to find work	Temporary Accommodation	No	Autism, Circulation Issues	PTSD^b^, Other (visiting psychologist for unspecified reason)
Laila	56	Other Ethnic group^a^	Asia (e.g., Middle East)	University Degree	Unemployed/ Unable to find work	Temporary Accommodation	No	Prefer not to say	PTSD^b^
Grace	57	Black British	Africa	University Degree	Unemployed/ Unable to find work	Temporary Accommodation	No	Tuberculosis (in recovery)	None reported
Rebecca	58	White other	Africa	University Degree	Unemployed/ Unable to find work	Shelter (e.g., Night Shelter)	No	Autism (Asperger’s syndrome)	PTSD^b^
Esther	54	Black other	Africa	Secondary (e.g., O level, GCSE)	Unemployed/ Unable to find work	Shelter (e.g., Night Shelter)	No	Chronic pain, Sickle Cell Anaemia	Depression
Gabriela	55	Other Ethnic group^a^	Latin America and the Caribbean	Secondary (e.g., O level, GCSE)	Part-time employment	Hostel	Prefer not to say	Chronic pain	Stress
Julia	44	White other	Europe	Prefer not to say	Prefer not to say	Hostel	Prefer not to say	Prefer not to say	Visiting psychologist
Emma	65	White British	Europe	Secondary (e.g., O level, GCSE)	Retired	Living with Friends and/or Family	No	Allergies	None reported

^a^The Other Ethnic Group included one Hispanic or Latin American woman and one woman describing herself as from the Middle East

^b^PTSD = post-traumatic stress disorder

GCSE = General Certificate of Secondary Education. Baseline corresponds to the participant’s first interview and data were self-reported by the participant using the demographic characteristics form. Eight of the nine women described experiences of sleeping rough but only one woman described currently sleeping rough when she was not able to access a night shelter

Additionally, two ICT class facilitators participated, providing insight into participants’ engagement with digital technologies, including digital health, and interactions with the facilitators and peers during ICT classes.

### Data collection

All interviews and observations were conducted by the first author who is an experienced qualitative researcher. The same researcher conducted all repeated interviews and observations with each participant, facilitating rapport and continuity across data collection.

#### Narrative interviews

Interviews adopted a narrative approach [43, 44, 53], using open-ended questions as presented above and minimal prompts to enable participants to guide the content and pace. Rather than following a fixed sequence of questions, participants were encouraged to share stories and experiences related to digital health in their everyday lives, with the interviewer using open-ended prompts and follow-up questions to facilitate narrative elaboration. Narrative approaches are increasingly used in health research due to their focus on person-centred care and understanding lived experiences of health and wellbeing [53].

A combined interview-observation guide was used to support both narrative interviews and participant observations. The guide included broad topics and prompts relating to digital technology use, digital health experiences, and interactions with services, as well as observational domains relating to digital technology use and social interactions within the study setting. Details of the interview-observation guide can be found in an earlier study which focused on how digital health can help or hinder older and middle-aged women to navigate paths through and out of homelessness [54]. The present study draws on the same longitudinal dataset and participants as [54] but specifically examines experiences of digital health through the lenses of inclusion health and eHealthism. For the purposes of the present study, the interview-observation guide was used flexibly and included open-ended prompts aligned with the study aims. Examples included: *How do digital technologies fit into your everyday life when it comes to your health or wellbeing? Can you tell me about a situation when you used these – what happened*,* and how did you feel about it? How have you used digital technology to interact with health or social care services*,* or with charities*,* community groups*,* or support organisations? Can you tell me about a few experiences and what they were like for you?*.

Baseline sociodemographic data (e.g., age, ethnicity, housing status) were collected during the first interview via a demographic characteristics form. The longitudinal design facilitated interviews at different time points, to check interpretations and follow up on topics and themes in subsequent data collections (Table 1) [44].

Narrative interviews were conducted one-to-one in a private room at the third-sector organisation. Refreshments were provided and a flexible, participant-led approach was adopted (e.g., offering breaks, easy-read materials). Interviews lasted between 26 and 130 min (mean: 52 min) and were audio-recorded and transcribed verbatim by the data collector. All transcripts, field notes, and quotations were pseudonymised prior to analysis, and participants were assigned pseudonyms to protect confidentiality.

#### Participant observations

Participant observations were used in combination with the interviews, inspired by the narrative-in-action approach [53]. Narrative-in-action views narratives as embedded in everyday actions and interactions, recognising that meanings are expressed and negotiated not only through spoken accounts but also through what people do in their daily lives and social contexts [53]. Accordingly, participant observations were used alongside narrative interviews to capture both narrated experiences and observed actions.

ICT classes were open to all individuals accessing services at the third-sector organisation. Consequently, class attendees included four participants from the narrative interviews, as well as other service users. During participant observations, the data collector focused primarily on the narrative interview participants who were present, while also observing their interactions with ICT class facilitators and peers. The data collector observed ICT classes with permission from all attendees, focusing on interactions related to digital technology and digital health use, including conversations, non-verbal communication, support received, peer-to-peer and peer-to-facilitator interactions. The ICT classes were more structured, and participants could choose to focus on practising a specific ICT task on one of the provided computers or via their personal digital device with support from an ICT facilitator. Participant observations were also embedded in the interviews, where participants could opportunistically engage in digital health activities that were relevant to their everyday life at the time of the interview (e.g., booking appointments, searching for health information). Field notes were written after each observation and included descriptive and reflective content [55]. Transcripts and field notes (collectively referred to as “the text”) were analysed using NVivo software (version 12).

### Data analysis

Data were analysed using a three-phase phenomenological-hermeneutic approach inspired by Ricoeur’s narrative and interpretation theory [44, 56], characterised by a hermeneutic spiral of iterative data collection and analysis [43, 57]. Three researchers (SNG, EM, PR) were involved in all stages of the analysis. The three researchers collaboratively engaged in the analysis in three stages: (1) naïve reading, (2) structural analysis, and (3) critical interpretation and discussion (sometimes referred to as comprehensive understanding). Naïve reading involved multiple readings of the transcripts and field notes in an open-minded way to gain an overall impression of the text as a whole [43, 44]. Structural analysis was used to confirm and refine the naïve reading by identifying units of meaning (what was said/observed) and significance (what it was about), coding data to initial themes and subthemes from the first data collection and comparing subsequent collections for continuity, change, or new themes and subthemes. Critical interpretation and discussion were used to contextualise themes and subthemes with relevant literature and theory, forming the discussion section. Ongoing discussions among the data collector and participants, as well as the wider research team, including the Women’s Advisory Board for Inclusion Health, helped to validate themes and subthemes, informing further data collection and analysis. Table 1 presents an overview of the data collection and analysis activities, including member checking which was performed with the participants in person at the recruiting third-sector organisation. Inspired by narrative methods, the purpose of returning to the participants in this way was less about validation and more about respecting the autonomy of the participants and their role in co-constructing narratives with the data collector [58]. Thus, the ongoing analysis and interpretation of findings were discussed with both the participants themselves and the Women’s Advisory Board for Inclusion Health.

### Ethical considerations

The study adhered to the Helsinki Declaration’s ethical principles [59] and was approved by the UCL Research Ethics Committee (No. 23885/001). Additional approval was obtained from the Swedish Ethical Review Authority (No. 2023-00211-01) to share anonymised transcripts for analysis with the research team at Marie Cederschiöld University College in Stockholm, Sweden. Participants received tailored information (i.e., detailed sheets, easy-read versions, or leaflets) based on literacy needs, emphasising the voluntary and confidential nature of the study. All participants provided written informed consent at the first data collection and verbally recorded informed consent at subsequent data collection sessions.

### Reflexivity and positionality

The interdisciplinary research team comprised of female researchers and research partners who contributed varied perspectives from occupational therapy (SNG), clinical psychology (PR), nursing and midwifery (AK, EM), expertise in informatics (JEL), as well as lived experience of homelessness (Women’s Advisory Board for Inclusion Health). The data collector brought prior experience with inclusion health groups and digital equity. She maintained a reflexive journal to document personal and methodological reflections, supporting transparency and trustworthiness, and recorded detailed information about the context and study process to be able to demonstrate the dependability and transferability of the findings given the study circumstances [60, 61].

To increase credibility and confirmability, we included triangulation of data collected through the narrative interviews and participant observations, prolonged engagement, and member checking [60, 61]. Recognising the co-constructed nature of narrative inquiry, the prolonged engagement helped to elucidate both the researchers’ and participants’ lived experiences, pre-understandings, complexities, and tensions [58]. One contributor in particular, a woman aged over 50, has been a member of the Women’s Advisory Board for Inclusion Health since 2020. Drawing on her lived experience of homelessness, violence, and substance use, she provided an in-depth interpretation of the analyses and advised on the salience of the findings as well as recommendations for inclusion health. She has contributed to other narrative research [62] and her name is not used due to her protected identity.

We generally use person-first language (e.g., women experiencing homelessness). However, identity-first language is used where this reflects preferences within specific communities or relevant literature, such as when referring to autistic people or autism.

## Results

### Study population

Table 2 presents the baseline sociodemographic characteristics of the nine women experiencing homelessness, aged from 44 to 68 years (mean [SD]: 55.9 [7.7] years). Most reported multiple physical and mental health conditions; only one maintained contact with family or relatives. Eight were born outside England, including one from the Middle East who declined to specify further. Sociodemographic data were not collected from the two ICT class facilitators as the focus was on the women experiencing homelessness’ interactions in the ICT classes.

### Naïve reading

Participants engaged selectively with informal digital health resources (non-institutional platforms such as YouTube and smartphone apps) and formal digital health resources (institutionally provided and regulated services such as National Health Service (NHS) websites, online resources, and telehealth). Access depended on relational support which was crucial yet unstable in the context of homelessness. While informal digital health offered resources for self-care and self-management, formal digital health sometimes deepened exclusion, particularly when perceived as impersonal or overly complex. Participants actively engaged with digital health, but engagement was shaped by personal values and preferences regarding care. Some resisted formal digital health resources, particularly telehealth services, when they were perceived as dehumanising substitutes for face-to-face interactions. Intersecting needs and circumstances, including memory difficulties, neurodiversity, mental health challenges, and housing instability, further complicated digital health engagement.

### Structural analysis

We identified three themes in relation to our study aims: (1) Taking care of my health on my own terms – with or without digital tools; (2) When digital tools are not enough – I still need support; and (3) Digital health can work – but it’s not the same as seeing someone face-to-face.

Participants’ accounts related to their everyday experiences of engaging with a range of formal and informal digital health resources as described in the findings, including telehealth services, online health information, self-monitoring tools, smartphone applications, and online peer support resources. Participants spoke broadly and fluidly about digital health for both physical and mental health purposes, differentiating between these only in specific contexts, as illustrated in the subthemes below. Where relevant, short background descriptions are included to situate participants’ narratives and highlight the intersectional nature of inequities.

#### Theme 1: Taking care of my health on my own terms – with or without digital tools

Participants expressed a sense of agency and resistance in managing their health, taking proactive steps to seek and engage with a range of digital health resources. This included both informal digital health resources, such as online health information seeking, peer support, recovery and wellbeing resources, and smartphone-based self-monitoring, as well as formal digital health resources, including telehealth appointments, NHS online services, and digital communication with health and social care services. However, engagement with digital health was shaped by participants’ personal values, preferences, and lived circumstances. Some participants actively resisted formal digital health resources when they perceived them as unsuitable for communicating complex health experiences or as misaligned with their needs and preferences for face-to-face care. Resistance was not described simply as non-compliance or digital exclusion, but as a values-based assertion of their preferences for either digital or face-to-face interactions.

##### Subtheme 1.1: Getting by alone – digital tools that support everyday life

Several participants commonly described using informal digital health resources to stay connected and to manage their everyday health and wellbeing. These became a “lifeline” during times of crisis or ill-health especially when this stopped them from going outside into society or attending face-to-face appointments, as one participant, Laila, explained:

Internet is the best thing because you can find out anything you need to know… with my condition, sometimes I’m not feeling well and I’m stuck indoors so I have to have our internet connected and it’s all about how to manage your life and health.

(Laila, Data collection 01, Interview)

In subsequent interviews, Laila expanded her use of online resources from information-seeking to more active monitoring and management of her health condition. Recalling a face-to-face appointment at the hospital where Laila was informed that her health condition is incurable and only manageable, she likened it to a “life sentence”. This led her to increasingly rely on informal digital health for active self-monitoring of her health and wellbeing. Like other participants, Laila questioned the accuracy of informal digital health, but she valued the insights it provided:

I use it [smartphone] for steps counting… heart rate… sometimes when I’m stressed, I will check my stress level and it’s something like a health check… blood glucose… blood pressure … you never know how accurate it is, but it still might give you a guide.

(Laila, Data collection 03a, Interview)

Participants emphasised the importance of online resources for accessing support for trauma and substance use disorders, including 12-step meetings, free therapy, and services tailored to women experiencing homelessness. This was described as having a “major impact”, helping them to prepare emotionally and practically for face-to-face healthcare appointments:

Self-care in YouTube. Oh yes, the NHS videos on YouTube, it shows the appointment, what to do at the appointment, what to expect… explaining step-by-step. I search as much as I can! Until I’m satisfied and relieved!

(Isabel, Data collection 02, Interview)

Over time, Isabel shared a more critical view that the effort and agency required for engaging with digital health was undermined by the challenging lived experience of homelessness:

It [digital health] requires a lot of energy, effort and willingness. In homelessness, there’s not willingness, there’s just self-abandon… because that’s the environment that she’s living in. So, there’s this little energy to do the daily basic things for yourself, which is, shower, right? Basic needs… That could be a time when technology you could use it to seek help, but you have got to have the willingness to use it.

(Isabel, Data collection 03a, Interview)

Other participants described using mobile phones or smartphones to record their medical history, track medications, and manage appointments. However, instability while experiencing homelessness, including frequent loss or theft of digital devices, undermined these strategies. One participant, Emma, reflected on the importance and responsibility of having a smartphone, which had been recently stolen:

I just had sort of everything in there [smartphone]. It was like my diary was in there. I log everything, medical appointments, medications, or whatever.

(Emma, Data collection 03a, Interview)

##### Subtheme 1.2: Being seen and heard – saying no to unwanted care

While many participants embraced informal digital health resources for self-management, others resisted formal digital health resources based on their personal values, cultural preferences, or distrust in the suitability of remote appointments or assessments when communicating about complex health experiences. One older participant with a migrant background, Esther, actively resisted telehealth and insisted on face-to-face care to more clearly explain her symptoms related to her Sickle Cell Anemia. Esther challenged the continued use of telehealth in a post-pandemic context but acknowledged the importance of being able to access her health records and documentation via the NHS App for administrative purposes related to her migration status:

Because when I refuse the telephone call, I was having pain in my right foot. Like, if I walk, I feel pain. I don’t only want to explain this on the phone. They say, ‘oh, you have to go for the blood test. Bla, bla, bla’. But the migrant centre requested for my medical reports and I have to prove this.

(Esther, Data collection 01, Interview)

Direct access to digital health records was experienced as supporting self-management by Esther and other participants, as it enabled them to obtain health information without relying on a health professional. However, this greater autonomy was accompanied by challenges in independently interpreting the meaning and implications of the information.

Another participant, Grace, also with a migrant background, favoured natural remedies and resisted mainstream medications based on her religion and upbringing on a farm in Africa. While Grace used the internet to search for natural remedies, she asked for face-to-face appointments to ensure that her values and preferences were heard and respected:

I don’t take medication. I have never taken half of paracetamol. I’m against it… I’m a Christian and I believe in natural, divine healing. So, when they call me on the phone I said, ‘No, I need to see someone because I need to explain why I don’t want it.’

(Grace, Data collection 01, interview)

In subsequent interviews, Grace’s values remained consistent with her resistance to using mainstream medications and preference for searching for natural remedies online. Grace reflected that she could better address her health needs and preferences if she lived in stable accommodation and had access to information to be able to search for remedies online:

When I started to Google it, it showed me so many things … if I was in my house, I would take only natural. I was brought up with herbs (making tutting sound). My parents are farmers. My dad, he would cook a big pot of herbs. Every morning, he would give me one cup to drink.

(Grace, Data collection 02, interview)

Many participants enacted resistance by questioning the suitability of telephone or online assessments for evaluating surgical outcomes or providing post-operative support, which they felt required face-to-face visual and physical evaluation, as expressed by one participant:

Can we have the assessment over the phone? How on earth you’re going to see the operation success?

(Laila, Data collection 03b, interview)

#### Theme 2: When digital tools are not enough – I still need support

Without traditional familial or friendship support networks, participants’ digital health engagement often depended on relations with key workers, ICT class facilitators, or staff from third-sector organisations, to access digital devices and for ongoing support to navigate digital health resources. These dependencies exposed tensions between the expectation of individual digital health engagement and systemic barriers for women experiencing homelessness. Participants rarely described digital health resources as being explicitly designed or adapted to meet the needs of women experiencing homelessness or other inclusion health groups. Digital health engagement was rarely individual or autonomous for most of the women, instead it was deeply relational and contingent on fragile support systems. Engagement with formal digital health was typically mediated through interactions with health and social care professionals and systems, while meaningful engagement with informal digital health often depended on support from third-sector organisations, key workers, ICT facilitators, and informal relationships.

##### Subtheme 2.1: I want to use digital health – but I can’t do it on my own

Ongoing relational support to access and use digital health was critical for participants living with intersecting needs while experiencing homelessness, such as memory issues, autism, and limited health and digital literacy. However, this support was often precarious and inconsistent, resulting in disruptions in their digital and digital health engagement. An older participant with memory issues with a migrant background, Evelyn, initially benefitted from support in ICT classes at a third-sector organisation. As support decreased over time, Evelyn struggled to maintain digital engagement independently:

My laptop, I get it at [Alternative homelessness provider]. I used to go there, a class… They don’t help me much now.

(Evelyn, Data collection 02, interview)

Similar to others, Evelyn had to adapt and seek support from several different services. She accessed a smartphone through a different third-sector organisation, although support about how to use it was limited and she perceived that it could be patronising at times. Evelyn described feeling expected to independently navigate digital health resources which may exacerbate other exclusion mechanisms linked to her lack of a support network, limited trust, dependency on support services, and ongoing health issues. Despite changing sources of support via a different third-sector organisation, digital literacy challenges and memory issues persisted:

Everything, in this one [smartphone]… but don’t ask me. I don’t get how to do it. I said, “Me God, what me know about this phone?”. It’s new and he [staff member at a third-sector organisation] told me about how to do all the things. I feel that I’m not doing it! Because of how he do it all. I can’t remember these things. The next day I call him. And he said, ‘Come, come back, let’s see’. I said, ‘No, I’m not coming back. I’m a big girl. I’m not supposed to’.

(Evelyn, Data collection 02, interview)

Participants were frequently observed struggling to use digital devices, both their own (e.g., phones, smartphones, and laptops) and those provided in ICT classes (e.g., PCs). A participant described the effort of learning digital skills as “pushing a stone up a hill”, reflecting the emotional and cognitive demand for those unfamiliar with digital technologies who were living with multiple health and social needs while experiencing homelessness. Participants often prioritised immediate basic needs, such as food, drink, shelter, and personal safety, over digital and digital health engagement. During an ICT class, Evelyn explained that she was hungry and thirsty. After the facilitator provided Evelyn with some food and drink, she was visibly more relaxed and engaged in the ICT class. The ICT facilitators emphasised the importance of tailored, one-to-one support:

The goal is to have about eight people per class but sometimes it can be preferable to have a smaller class so that there is more one-to-one support and attention for the women. (Observation ICT class 2)

##### Subtheme 2.2: It makes me feel alone – digital care can shut you out

Digital health placed expectations on individuals to manage their health independently, but participants often perceived this as impersonal, isolating, or insufficiently responsive to their complex needs. The lack of personal connection in digital interactions compounded feelings of exclusion, with some disengaging entirely from digital health resources. Rebecca explained that she had been locked out of her NHS online account but expressed minimal interest in resolving it since she appreciated walk-in services for healthcare, which may also reflect the availability of other forms of support:

Well, when I need healthcare, I go to the [London borough]… what’s it called, [London borough medical practice]. And they’re very good, and you can walk in when you like. So, I don’t really have to make appointments using umm… although I’ve been locked out of the NHS online. I don’t know why, I got a message about a month or two ago. And I haven’t bothered. I don’t know. There’s some error or something.

(Rebecca, Data collection 02, interview)

Others described a sense of abandonment, exclusion, and frustration when experiencing barriers to accessing digital health, especially if there was an expectation that it would be universally easy-to-use:

The NHS put me out because I was garbage. And then, after I left, I began to recover from all the distress. I didn’t have online access. And I didn’t have this phone either. They told me to contact them to arrange an appointment online. But as I was on the website it didn’t work… Like they make you think this is the website, it should be very simple for everybody to use. But I need to read many things and I’d given up.

(Julia, Data collection 01, Interview)

Participants also expressed isolation using digital health resources as they missed social connection:

I can’t function on the telephone because it’s just me and this voice. I feel so alone with it. I need to see someone face-to-face to explain how I am feeling.

(Esther, Data Collection 01, Interview)

Participants valued the social aspects of the ICT classes and appreciated opportunities for relational interaction and support. A participant described them as crucial to her health and wellbeing:

I like doing the computer classes because it is important! If I stay home, I will get sick.

(Observation ICT class 7)

##### Subtheme 2.3: Passed around - no one sees the whole picture

Participants encountered fragmented care pathways across both digital and face-to-face services. However, digital health resources often added further navigation work, requiring participants to coordinate referrals, repeat information, and interpret health information across multiple services while managing complex and intersecting health and social needs. Isabel, an autistic participant with a migrant background, described navigating fragmented care pathways across multiple services. While these challenges were not limited to digital health, digital communication and online information seeking formed part of how she navigated referrals, appointments, and unfamiliar medical terminology:

They did send me a text and then I call them back but it’s always like, ‘Oh, you need to speak to this person,’ and then they’re not there, so I have to call again, and it’s just a mess!… I went to the hospital and then they send me back to the GP because they say it’s not their thing, and then the GP says, ‘No, you need to go back,’ and I’m like, ‘What am I supposed to do?’. It’s just going round in circles!… ‘What on earth means [name of virus]?’. I look that online because I didn’t know what they were talking about.

(Isabel, Data collection 02, Interview)

Another participant, Emma, who had prior administrative work experience using digital technologies, expressed her frustration with being repeatedly referred to emergency services through the “E-doctor” system, a formal digital health resource, rather than receiving the follow-up care she felt that she needed:

I’ve tried, the GP sent me the E-doctor. And when I complete everything there, it’s just phone 999 or go to A&E [Accident and Emergency department at a hospital], which is ridiculous… I spent an hour talking to the receptionist… another hour with a nurse… they said that the GP will call you*.*

(Emma, Data collection 03a, interview)

Participants reported having to repeatedly share their health histories and navigate multiple digital and face-to-face services, which they perceived as burdensome and poorly suited to the complexity of their circumstances. This was perceived as failing to accommodate diverse experiences and complex needs, requiring participants to repeatedly explain and coordinate their care across multiple services. Participants described how more coordinated responses across health and social services were often only triggered once a situation had reached a crisis point, such as a hospital admission. Esther described how health and social care professionals worked together to secure appropriate accommodation following her hospital admission:

And the hospital nurse said, “Ok, with your condition that place is not good for you because you are vulnerable. We won’t let you go back there [shelter] and we will not let you go on the streets. We will make sure that we find you a suitable place before we can discharge you.” It was the social worker and hospital that find that place for me.

(Esther, Data collection 02, Interview)

#### Theme 3: Digital health can work – but it’s not the same as seeing someone face-to-face

The relevance of digital health for participants was situational and contextual, shaped by immediate needs, access to digital devices, and trust in digital health resources. Participants described using both formal and informal digital health resources. Participants creatively blended these forms of digital health resources for immediate support and to manage their health and wellbeing routines. However, digital health was not perceived as a panacea, often failing to replace the human connection of face-to-face care or proving unreliable due to technical problems or when systems malfunctioned.

##### Subtheme 3.1: I get support online – through videos, apps and searches

Informal digital health resources through everyday digital technologies, including YouTube or smartphone apps, were often perceived as more accessible and reliable than formal digital health resources. Participants described using YouTube as a source of comfort during times of distress or crisis, turning to humorous or informative content to feel less isolated:

I’m feeling suicidal then I go to YouTube and watch something funny or watch something informative and that takes me out of isolation.

(Isabel, Data collection 01, interview)

Participants described using a range of informal digital health resources, including online health information, NHS videos, smartphone applications, and online searches relating to nutrition, fitness, and wellbeing. These resources provided immediate support for mental health, nutrition, fitness, and overall wellbeing routines in everyday life, particularly when living in more stable environments that supported routine-building. Over the course of the study, Gabriela, a participant with a migrant background, secured more stable accommodation with access to a communal kitchen which enabled her to prepare healthier food and drink and focus on her interest in nutrition. Gabriela integrated formal digital health resources, such as online nutrition information and videos from the NHS website, with smartphone apps to create vision boards and guide her meditation and fitness routines:

Gabriela: I do every day on my phone. I’m writing also on the app every day. [The participant shows the interviewer her phone with photos of vision boards] Well, when I wake up, I do my vision board, meditation… I do workout for full body.

Interviewer: Has there been a difference [since moving to new accommodation with communal kitchen access]?

Gabriela: Yesssss! It’s more easy for me. I can do properly my smoothies. I feel freedom. You know, for me it’s important the nutrition.

(Gabriela, Data collection 02, interview)

##### Subtheme 3.2: Face-to-face care matters – especially for mental health

Despite their regular use of digital health, participants consistently emphasised that these were not substitutes for face-to-face interactions, especially regarding mental health. The relational and embodied aspects of face-to-face interactions were considered essential for understanding and communicating about trauma and complex needs:

Mental health for me, I always prefer face-to-face because it relies on words … your expression, your body language, the struggle, it’s all there.

(Laila, Data collection 03a, Interview)

Participants appreciated access to free online therapeutic resources, with one participant describing this as “lifesaving”:

Access to free therapy because when I’m feeling down then I can go online and it’s amazing because it’s all there and it has saved my life. It’s instant gratification but then there’s no time for patience.

(Isabel, Data collection 01, interview)

In subsequent interviews, Isabel highlighted that her preferences for digital health were contextual. Isabel resisted online group trauma workshops as a full substitute, maintaining a preference for face-to-face, one-to-one therapy:

Trauma workshops online… I don’t want these workshops as a replacement. My aim is one-to-one, face-to-face therapy.

(Isabel, Data collection 02, Interview)

##### Subtheme 3.3: I trust digital tools - but only sometimes

Trust in digital health was not static; it fluctuated based on participants’ previous experiences, emotional states, and situational needs. The same participant may perceive different forms of digital health and broader digital communication associated with health, housing, and social care services as less judgmental, more flexible, and easier to control in some situations. At the same time, participants could also feel frustrated or cautious about sharing personal data in other contexts. Another participant, Laila, explained that being able to edit or delete messages on digital platforms, or those related to her local council, housing, and social services, helped mitigate feelings of embarrassment or judgement:

… the magic about the technology, you can actually change your words, you can delete it and you can’t do this with human being communication. And even if you mispronounce the word, you wouldn’t feel embarrassed because the technology doesn’t judge you.

(Laila, Data collection 03a, interview)

Julia, a participant with a migrant background and complex mental health needs, described how difficulties completing an online NHS consultation form used to request healthcare support compounded her sense of isolation, contrasting this with the aspirational sense of control she experienced while playing a computer game:

I just love to play it [Sims], it’s amazing because I can control it, you know, and it helps me think about how life could be. But then with the health stuff, it’s different. I tried filling in the online form but it was just too much, I couldn’t do it properly because it kept crashing or something, I don’t know. As I was on the website it didn’t work, and I felt so alone trying to figure it out, like nobody was there to help me when I needed it most.

(Julia, Data collection 01, Interview)

Overall, participants described profoundly negative face-to-face interactions with health and social care professionals. These experiences shaped how participants engaged with digital health, with some valuing the distance, flexibility, and perceived lack of judgement offered by digital communication, while others experienced digital health as exacerbating feelings of disconnection and isolation.

## Discussion

To our knowledge, this is among the first studies to explore digital dimensions of inclusion health through the critical lens of eHealthism. It is also novel in being one of the few qualitative longitudinal studies focusing on women experiencing homelessness [2, 41], particularly in the under-researched area of their lived experiences of digital health. The main findings based on the three themes identified in the analysis are critically interpreted and discussed below.

### Critical interpretation

Through a Ricoeur-inspired phenomenological approach, we moved from participants’ lived experiences to a critical interpretation that situates these experiences relative to the dominant logics of Healthism, which we extend in this study to eHealthism. We use eHealthism to capture how contemporary digital health discourse often assumes that individuals can and should become active, engaged users of digital health technologies [9, 10, 12]. Our analysis problematises these assumptions by highlighting how such expectations may overlook the social and structural determinants that shape access to, engagement with, and use of digital health.

A key finding was that participants often used informal digital health resources, such as YouTube, meditation apps, or search engines, to inform themselves about health-related issues and manage their wellbeing. These informal digital health resources were perceived as more accessible and relevant than formal digital health resources. However, participants’ engagement with these informal digital health resources was shaped by access to material resources and stable living conditions, which created the time, capacity, and sense of safety needed to engage with digital health. Housing precarity, food insecurity, and safety concerns often limited opportunities for such engagement. Participants used metaphors such as “lifeline,” “life-sentence,” and “lifesaving” to describe the complex and at times contradictory role of digital health in their everyday lives. Such findings are consistent with prior research on the fragility of digital inclusion in women experiencing homelessness, where engagement is not linear or sustained but rather it is interrupted and shaped by everyday crises [4, 5]. Notably, participants’ use of digital health resources was practical, strategic, and grounded in immediate needs. Thus, undermining the eHealthist assumption that limited engagement with digital health primarily reflects individual shortcomings, such as a lack of motivation, willingness, or capacity to engage, rather than the social and structural conditions shaping opportunities for engagement with digital health [39].

Instead of reflecting passive disengagement, participants’ resistance to formal digital health resources, particularly telehealth services and online mental health resources, was a deliberate and meaningful assertion of autonomy. For many, especially in relation to mental health needs, formal digital health resources failed to offer the relational and embodied forms of communication they valued. Eye contact, body language, and therapeutic presence were described as central to care experiences. Face-to-face care was not just preferred, but seen as essential, particularly for mental health care when communicating complex or trauma-related issues. Resistance in this context may be interpreted as a form of care in itself, as a way to reclaim control over how, where, and with whom health decisions are made. Williams et al. (2024) identified similar resistance and paradoxical tensions between connection and invisibility, whereby women experiencing homelessness sold the budget feature phones provided by formal homeless support services in the UK, in order to meet their immediate needs [5]. The individual responsibility to become a competent, autonomous digital health consumer may not always align with the everyday priorities of women experiencing homelessness and other inclusion health groups. Participants actively challenged digital health resources that did not accommodate their preferences or complex needs, reshaping their health narratives in ways that defy dominant eHealthist discourses which assume digital engagement as inherently empowering [39].

Participants’ digital health engagement was at times entangled with their experiences of trauma. Many described digital mental health resources and services, including online therapy, trauma workshops, telehealth encounters, and online wellbeing resources, as inadequate for addressing trauma and complex mental health issues. In digitally mediated encounters, participants described feeling unseen, unheard, or even dehumanised. These experiences were linked to the perceived absence of embodied and relational aspects of care, including non-verbal communication, emotional connection, and opportunities to communicate complex experiences of trauma and vulnerability. This corroborates concerns raised in research with people experiencing homelessness that describes experiences of dehumanisation and discrimination in healthcare settings more broadly, and the need for structural competent approaches, such as street medicine teams which meet inclusion health groups where they are at and in turn foster a greater sense of trust [28]. While some earlier studies highlight the benefits of telehealth [24], and positive perceptions and acceptability of digital mental health among people experiencing homelessness [36], including youth [17] and older adults [20], our findings highlight the need to critically reassess these claims within specific contexts, particularly among women experiencing homelessness and those living with complex trauma. Earlier research among older people experiencing homelessness indicates that perceptions of telehealth for physical health depended on video or audio-only modality [20]. However, in the present study, even video modalities were questioned by some as insufficient substitutes for embodied, face-to-face interactions, particularly for women experiencing homelessness who may also be living with complex trauma. Trauma treatment should ideally begin once safety, housing, and a supportive network are in place. Our findings align with research emphasising the importance of recognising and responding to gendered and trauma-informed care for women experiencing homelessness [37]. Moreover, our findings build on existing guidelines calling for trauma-informed care approaches in service delivery by emphasising its relevance in digital health services for women experiencing homelessness [25].

Importantly, participants’ digital health practices were embedded in relationships. Consistent with earlier research [4, 15], participants reported that lack of secure access to digital devices impeded their engagement with digital health, underscoring eHealthism’s neglect of social determinants [39, 63]. Exposure to violence or abuse can limit women’s access to digital devices, often controlled or monitored by perpetrators, making digital security important for women to feel safe to engage with digital health resources. Our findings highlight the importance of sustained access to digital devices, alongside ongoing support, guidance, and training. Notably, participants rarely experienced formal digital health resources as explicitly designed or adapted to meet the needs of themselves or other inclusion health groups. Instead, third-sector organisations played a crucial role not only in providing access to devices, but also in offering ongoing, person-centred support and in fostering social and physical environments that enabled engagement over time, echoing findings from research with youth experiencing homelessness [64].

In this study, inclusive and responsive practices were primarily evident in the relational interactions provided by third-sector organisations and ICT facilitators, rather than in the design of digital health resources themselves. Across the participant observations and accounts, ICT facilitators adopted a holistic approach that attended to the participants’ broader circumstances and immediate needs, such as food, drink, emotional reassurance, and one-to-one assistance, before introducing ICT. Such practices align with principles of trauma-informed care, which emphasise safety, trust, and responsiveness to lived experience. rinciples of trauma-informed care are particularly relevant given the participants’ experiences of violence, instability, and trauma. These relational approaches appeared to enable engagement with digital health in ways that technology-focused interventions alone did not. However, such practices remain largely overlooked in digital health research and policy [12, 15]. Current digital health research and policy tends to reinforce top-down digital inclusion initiatives that focus narrowly on device access or skill acquisition, while neglecting the broader social and emotional conditions necessary for meaningful engagement. Thus, the findings suggest that digital health research and policy would benefit from greater recognition of trauma-informed approaches in supporting engagement among women experiencing homelessness and potentially other inclusion health groups.

Our findings build on earlier research among other populations, such as low-income older adults [65], which suggest that digital literacy is more than a static, individual attribute as it is also relies on relational and contextual aspects, particularly to maintain long-term benefits. Extending critiques of eHealthism [39, 63], our findings suggest that access and use of digital health depend on care infrastructures, including third-sector organisations, ICT facilitators, and ongoing one-to-one support, which are frequently absent or undervalued in the context of homelessness. Gender disparities in digital literacy and education, in addition to cultural and social norms may limit women’s abilities to use digital health and opportunities for ICT training [13]. This is especially salient for the women living with intersecting physical and mental health issues, including two of the older women who had memory issues and required ongoing practical support and emotional reassurance to engage with digital health resources in a way that was perceived as relevant rather than patronising. This finding reflects existing research revealing high rates of cognitive impairment [34] and ADRDs [33] among people experiencing homelessness, as well as autism [32]. While our study is based on a small and purposive sample, several participants identified as living with (or suspected they lived with) cognitive impairment and/or neurodivergence, which, when layered with trauma, migration status, or mental health issues, made digital engagement particularly challenging. This substantiates a systematic review [3] highlighting the impact of intersectionality on service-engagement and help-seeking across different women in inclusion health groups, including women experiencing homelessness. Our findings extend the earlier systematic review [3] by demonstrating how intersecting vulnerabilities, including cognitive impairment, neurodivergence, trauma, migration-related experiences, and mental health challenges, shape engagement with digital health as well as conventional face-to-face services. These findings reinforce the need for digital health services that are responsive to the diverse and intersecting needs of women experiencing homelessness rather than assuming a one-size-fits-all approach to digital inclusion.

Finally, several participants shared experiences of cultural and epistemic dissonance. In other words, a clash between the competing epistemologies of some of the participants’ own knowledge and values around health (e.g. holistic, relational, culturally grounded) and those underpinning formal digital health (e.g. remote, data-driven, biomedical). This was evident in the way one participant with a migrant background preferred traditional natural remedies and sought face-to-face appointments to explain her choices. This challenges the universalist logic of digital health, which may overlook culturally embedded health practices and contextual relevance of digital technologies [64]. Such moments reveal a tension between the normative assumptions of digital health (i.e., rational choice, efficiency, individual responsibility) and the situated, diverse ways that women experiencing homelessness understand and care for their health and wellbeing [2–4]. Thus, our findings reinforce calls from scholars such as Helsper (2022) [63] and others [39] to interrogate the values embedded in digital health resources and their tendency to reproduce rather than redress health inequities.

### Recommendations for inclusion health

**Fig. 1:**
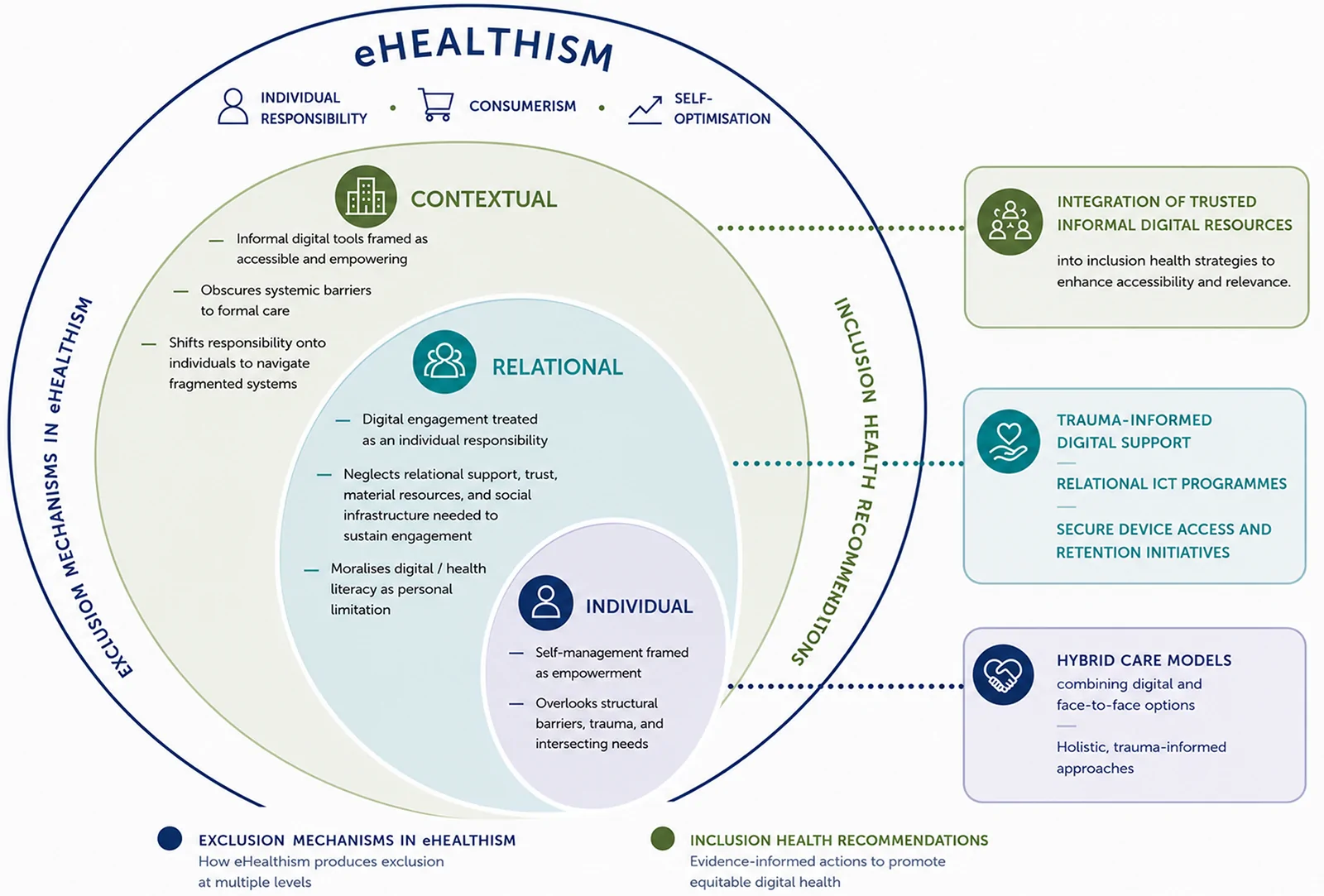
Multi-level exclusion mechanisms in eHealthism and corresponding recommendations for inclusion health

Our findings suggest a need to critically examine eHealthist assumptions within inclusion health. While digital health research and policy often promote access, self-management, and optimisation, these framings should also recognise the social and structural conditions that shape opportunities for engagement. Participants demonstrated considerable agency in their enagegment with digital health, but this agency was exercised within constraints related to housing instability, poverty, trauma, health conditions, and access to relational support. Digital health is not merely about access or literacy but also about relationality, trust, and context. Table 3 presents our themes and key findings, and maps these to a three-layer conceptual framework of exclusion mechanisms in eHealthism, with corresponding recommendations for inclusion health. Our conceptual framework is comprised of individual, relational, and contextual layers. Building on our critical eHealthist interpretation, we discuss the recommendations for inclusion health in the following section fig 1.

**Table 3:** Conceptual framework of exclusion mechanisms in eHealthism and recommendations for inclusion health

Layer	Theme	Key finding	eHealthism interpretation	Recommendations for inclusion health
Individual	Taking care of my health on my own terms – with or without digital tools	Women resisted formal digital health (e.g., telehealth) as substitutes for face-to-face care, especially when expressing complex mental health needs, such as trauma.	eHealthism emphasises individual responsibility, consumerism, and self-optimisation. While framed as empowerment, it overlooks social and structural barriers and the complexity of intersecting needs.	Develop hybrid care models integrating digital and face-to-face options, to promote holistic and trauma-informed care.
Relational	When digital tools are not enough – I still need support	Digital health engagement was relational and contingent on others, especially third-sector organisations, for access and ongoing support.	eHealthism’s focus on individual responsibility overlooks relational needs and infrastructure that sustain digital engagement, often moralising digital/ health literacy issues as a personal limitation.	Train staff in trauma-informed digital support and provide relational ICT programmes. Implement secure device access and retention programmes.
Contextual	Digital health can work – but it’s not the same as seeing someone face-to-face	Women used informal digital health resources but resisted formal digital health resources when these were misaligned with their everyday lives, values, and care preferences.	eHealthism promotes informal digital health resources as accessible and empowering, but this can medicalise everyday distress and shift responsibility onto individuals, ignoring systemic barriers to formal care.	Integrate trusted informal digital resources into inclusion health strategies to enhance accessibility and relevance.

#### Individual layer

First, participants’ preference for face-to-face care delivery, especially when managing complex mental health needs, indicates a need for choice regarding hybrid care models that may integrate digital and face-to-face options, whereby digital health is not considered a substitute but rather a supplement to face-to-face service delivery. To avoid reproducing eHealthist assumptions of individual responsibility, consumerism, and self-optimisation, such care models should be grounded in trauma-informed approaches to better respond to the complex and intersecting needs of women experiencing homelessness.

#### Relational layer

Second, participants’ digital health engagement was often supported by relationships with peers, health and social care professionals, and especially third-sector organisations. This underscores the importance of relational infrastructures, especially for women experiencing homelessness, who may face challenges related to digital literacy and who may not have conventional familial relationships or support networks. Inclusion health initiatives would benefit from supporting trauma-informed ICT programmes, including relevant staff training, and promoting consistent access to secure digital devices, not only for initial engagement but for ongoing use.

#### Contextual layer

Finally, participants frequently used informal digital resources such as YouTube, apps, or search engines, as they found them more accessible than formal digital health resources which were sometimes misaligned with their intersecting needs. While eHealthism positions such resources as empowering solutions to improve health engagement, it risks placing systemic responsibilities onto individuals. Inclusion health strategies should prioritise contextually adapted digital health approaches that respond to diverse and intersecting needs, including trauma, migration-related experiences, cognitive impairment, neurodivergence, housing instability, and varying preferences for digital and face-to-face care, to avoid imposing one-size-fits-all solutions that reinforce exclusion.

Together, these recommendations point to the need transcend a narrow focus on access and literacy driven views of digital health. Instead, we propose that inclusion health should embrace a more nuanced, equity-oriented framing of digital health, to recognise the importance of relational infrastructures and embed digital health in trauma-informed, hybrid, and contextually relevant care models. The findings indicate that digital health is best positioned as a flexible care option, supported by relational continuity and trauma-informed practice, rather than as the default route to care.

### Strengths and limitations

This study was strengthened by its longitudinal design, which involved collecting narrative and observation data repeatedly over time. The data collector spent extended time in the participants’ environments, meeting service users, and attending activities such as ICT classes. This type of extended engagement has been utilised in earlier homelessness research [41] and helped to normalise the data collector’s presence for participant observations [55]. Ricoeur’s (1984) distinction between chronological time (the sequential passage of events) and narrative time (how events are reconfigured into meaningful stories) [56] is particularly salient for women experiencing homelessness. Trauma, housing instability, and social exclusion often disrupt chronological continuity, fragmenting experiences, and creating temporal disorientation. By utilising narrative-in-action [53], a combination of repeated narrative interviews, participant observations of individual digital health activities, and group ICT classes, participants were able to reconfigure these ruptures as part of a process of meaning-making in relation to their digital health experiences.

The iterative process of data collection and analysis was supported by triangulation and member checking, enabling the data collector to co-construct narratives with participants and refine interpretations over time. NVivo software facilitated a structured approach to data management, collating interview transcripts, field notes, and reflections that informed the analysis process. These steps enhanced the trustworthiness and credibility of the study [60, 61]. Collaboration with an interdisciplinary team of researchers as well as experts with lived experience of homelessness facilitated critical discussion of analyses and interpretations. This collaborative approach supported more rigorous scrutiny of findings and enhanced the relevance to participants’ lived experiences. While digital health may be analytically distinguished into different technologies and service modalities, participants spoke more generally about “digital tools” or “digital health tools”; therefore, we adopt this terminology to remain close to participants’ narratives. An implication of this approach is that our findings reflect participants’ everyday experiences of digital health as a broad phenomenon rather than experiences associated with specific technologies or service modalities. Accordingly, the study was not designed to compare or evaluate particular forms of digital health (e.g., telehealth, mobile health applications, online health information resources, or electronic health records), and we cannot draw conclusions about differences in experiences across these technologies. Future research should explore how women experiencing homelessness engage with and perceive different digital health technologies and modalities.

A potential limitation is that ICT facilitators were not interviewed for this study. Thus, the findings do not capture facilitators’ perspectives on participants’ engagement with digital health resources. Instead, facilitators were included indirectly through participant observations of interactions between participants and ICT facilitators during ICT classes. While the inclusion of facilitator interviews may have provided additional contextual insights and enabled data triangulation, the decision not to interview facilitators was intentional. This approach sought to centre the perspectives of women with lived experience of homelessness, avoid diluting participant accounts through professional interpretations, and address epistemic injustices that can arise when professional perspectives are prioritised over lived experience expertise [66]. Consequently, the findings foreground participants’ own understandings and experiences of digital health engagement, which aligns with the aims of the study.

Inclusion health groups, such as women experiencing homelessness, have often been described as a hard-to-reach, transient, or “hidden” [46]. More recent scholarship, however, challenges such labels and argues that responsibility lies with researchers and institutions that have historically struggled to reach and engage these groups. In this study, recruitment was facilitated through a third-sector homelessness organisation, with staff supporting the identification of potential participants. This approach was appropriate given the study’s aims of understanding women’s everyday encounters with health and social care systems, including interactions with third-sector services.

A strength of this approach was the close collaboration with the recruiting organisation, which supported participant engagement and retention throughout the longitudinal study. However, recruiting through a single specialist homelessness service may have limited the breadth of perspectives included, as women with little or no contact with such services were less likely to be represented. In addition, all but one participant was born outside the UK. While participants represented diverse migration backgrounds, enriching understanding of the intersections between homelessness, migration, and digital health, the prominence of migrant experiences may limit the transferability of the findings to women experiencing homelessness who have longstanding familiarity with the UK health and welfare systems. Participants’ experiences of digital health may have been shaped not only by homelessness but also by factors associated with migration, including differing levels of familiarity with the UK healthcare system, previous experiences of healthcare in other countries, and language or cultural barriers to accessing services. Recruiting across a wider range of services may have increased the likelihood of including more UK-born women experiencing homelessness and broadened the range of experiences captured. Nevertheless, the purpose of this narrative study was not statistical representativeness or generalisability. The adequacy of participant numbers was guided by the concept of information power [50]. Consequently, participants contributed substantial information relevant to the study aims, supporting the adequacy of the participant group for this narrative inquiry. The transferability of these findings should be considered in relation to comparable inclusion health contexts in high-income settings, acknowledging the influence of the UK service context and the participating third-sector organisation.

## Conclusions

This study highlights how women experiencing homelessness engage with digital health in complex, often contradictory ways. Our findings underscore the nuanced engagement of women experiencing homelessness with digital health. On the one hand, digital health provided participants with access to various health and wellbeing resources without some of the barriers of face-to-face services. On the other hand, digital health exacerbated pre-existing health and digital inequities and intensified a sense of marginalisation among the participants who are already at risk of social exclusion. While personalisation, autonomy, and individual empowerment have been highlighted in earlier research, all participants in the present study relied on support from others, including peers, health and social care professionals, and especially from individuals working at third-sector organisations. The role of third-sector organisations in bridging service gaps is frequently overlooked in research, but our findings revealed that these organisations provide digital devices as well as the necessary ongoing training and support to use them for digital health and other purposes. In the context of inclusion health, relational dimensions of digital health are crucial to ensuring inclusive, trauma-informed digital health interventions for women experiencing homelessness.

Through the critical lens of eHealthism, our findings contribute to health equity by highlighting the need for digital health to recognise intersecting social and structural determinants and relational dimensions that shape, and are shaped by, women experiencing homelessness’ engagement with digital health in their everyday lives. Personal responsibility for health through digital health cannot be assumed during homelessness; meeting basic needs and safety must come first before placing the responsibility of care on individuals. Creating the conditions that enable meaningful engagement with digital health, including access to housing, safety, material resources, and ongoing relational support, requires investment in health and social care services, third-sector organisations, and wider social support systems. Thus, our study calls for digital health initiatives to address these complexities to promote equitable and meaningful access for women experiencing homelessness and other inclusion health groups. 

## Supplementary Information

Below is the link to the electronic supplementary material.

Supplementary Material 1

## Data Availability

The dataset generated and analysed during the current study is not publicly available due to data governance considerations but is available from the corresponding author on reasonable request.

## Abbreviations

ADRD: Alzheimer’s disease and related dementia
A&E: Accident and Emergency department at a hospital
ETHOS: European Typology of Homelessness and Housing Exclusion
FEANTSA: European Federation of National Organisations Working with the Homeless
GCSE: General Certificate of Secondary Education
GP: General Practitioner
ICT: Information and Communication Technology
NHS: National Health Service
NIHR: National Institute for Health and Care Research
PC: Personal Computer
PTSD: Post-Traumatic Stress Disorder
SD: Standard Deviation
SRQR: Standards for Reporting Qualitative Research
UK: United Kingdom
WHO: World Health Organization
